# Political Orientation Moderates the Relationship Between Climate Change Beliefs and Worry About Climate Change

**DOI:** 10.3389/fpsyg.2020.01573

**Published:** 2020-07-16

**Authors:** Thea Gregersen, Rouven Doran, Gisela Böhm, Endre Tvinnereim, Wouter Poortinga

**Affiliations:** ^1^Department of Psychosocial Science, Faculty of Psychology, University of Bergen, Bergen, Norway; ^2^Centre for Climate and Energy Transformation, Faculty of Social Sciences, University of Bergen, Bergen, Norway; ^3^Department of Psychology, Inland Norway University of Applied Sciences, Lillehammer, Norway; ^4^Department of Administration and Organization Theory, Faculty of Social Sciences, University of Bergen, Bergen, Norway; ^5^Welsh School of Architecture, College of Physical Sciences and Engineering, Cardiff University, Cardiff, United Kingdom; ^6^School of Psychology, Cardiff University, Cardiff, United Kingdom

**Keywords:** worry, beliefs, causes, impacts, climate change, political orientation

## Abstract

Public perceptions are well established as a key factor in support for climate change mitigation policies, and they tend to vary both within and between countries. Based on data from the European Social Survey Round 8 (*N* = 44,387), we examined the role of climate change beliefs and political orientation in explaining worry about climate change across 23 countries. We show that belief in anthropogenic climate change, followed by expectations of negative impacts from climate change, are the strongest predictors of worry about climate change. While the strength of the association between political orientation and worry about climate change varies across countries, self-positioning further to the right of the political spectrum is associated with lower levels of worry in most of the countries included in the analysis. We further show that political orientation moderates the relationship between climate change beliefs and worry. While increased confidence in the anthropogenic nature of climate change and expectations of negative impacts are both associated with increased worry across the political spectrum, the relationship is weaker among right-leaning as compared to left-leaning individuals. Notably, the main effect of political orientation on worry about climate change is no longer statistically significant when the interaction terms are present. Finally, a relatively small amount of the explained variance in worry is attributable to differences between countries. The findings might inform strategies for climate change communication in a European context.

## Introduction

Public acceptability is recognized as a key factor for the successful implementation of measures directed at tackling climate change (de Coninck et al., 2018). Understanding how individuals perceive climate change can thus be central to mobilizing support for climate policies. Previous research shows that the extent to which individuals worry about climate change can vary within countries in regards to social, cognitive, and cultural factors (Van der Linden, 2017), but also across countries when considering the overall level of worry expressed by the general public (Poortinga et al., 2019). In addition, Poortinga et al. (2019) demonstrated that the predictive strength of socio-political and demographic variables in explaining concerns about climate change also differs across countries and regions. The present study builds upon that research to shed further light on how public perceptions of climate change interact with political orientation in a European context. In particular, we will focus on the relative importance of and interactions between climate change beliefs and political orientation in explaining worry about climate change.

Worry is one of several measures used to study climate change risk perception, sometimes interchangeably with the concepts of concern, perceived seriousness, and perceived risk. Van der Linden (2017) proposes that personal worry, generalized concern, perceived severity, and likelihood ratings are all components of a “hierarchy of concern,” and that personal worry is the preferred indicator if the goal is to understand the association with behavior and/or policy support. In line with this, Van der Linden et al. (2019) found that personal feelings of worry are associated with higher levels of support for public action on global warming and that this association is stronger for worry than for more cognitive judgments. Smith and Leiserowitz (2014) reported that worry is a far more important factor in support for climate mitigation policies than are sociodemographics, cultural worldviews, and other discrete emotions such as hope or anger. Consequently, identifying what makes individuals worry about climate change may help to provide a better understanding of public support and engagement with the issue.

One factor that is often associated with people’s level of worry is their beliefs about the causes and possible impacts of climate change, sometimes referred to as mental models (Bostrom, 2017). Previous research has shown that people are more likely to report concern about climate change when they think that humans are responsible for causing it (Lee et al., 2015; Shi et al., 2016), and to be more willing to engage in pro-environmental behaviors and to pay for policies when they think the consequences of climate change will be severe (Mayer and Smith, 2019). Beliefs about consequences for humans have been found to be central to environmental risk perceptions (Böhm and Pfister, 2001) and several experimental studies support that worry is an especially likely emotional reaction when focusing on possible negative consequences of environmental risks (Böhm, 2003; Böhm and Pfister, 2005). Tobler et al. (2012) reported that, out of several types of climate change knowledge, knowledge about causes was most strongly related to climate change concern. Furthermore, Böhm and Pfister (2017) found that human-caused risks are more strongly related to moral blameworthiness and emotions such as outrage than are natural risks, which suggests that causal attributions are important for evaluations and emotions relating to environmental risks, including climate change.

Another factor known to be associated with climate change perceptions is political orientation, which, according to McCright et al. (2016), constitutes one of the most important and consistent predictors of climate change perceptions such as worry and concern. A common approach to measuring political orientation is to ask people to position themselves on a liberal versus conservative (in the United States; e.g., American National Election Studies) or a (political) left versus right (in Europe; e.g., European Social Survey) dimension. Research has found that left-leaning or liberal individuals are more likely to believe in the reality and anthropogenic nature of climate change, and to be worried about it, than those who identify themselves as right-leaning or conservative (for a review, see McCright et al., 2016).

Studies in which individuals place themselves on a liberal-conservative continuum support this relationship for belief in anthropogenic climate change (Hornsey et al., 2016) and environmental concern (Cruz, 2017; Leiserowitz et al., 2019). Most of these studies were conducted in the United States and report small- to medium-sized associations. Measuring political orientation on a left-right continuum in an international context, Kvaløy et al. (2012) as well as McCright et al. (2015) found that left-leaning individuals are more likely to perceive climate change as a serious problem. Doran et al. (2018) found that political orientation predicted support for climate policies, even when controlling for consequence beliefs and moral concerns about climate change. And Poortinga et al. (2019) found a clear and highly consistent negative association between right-leaning political orientation and climate change concern using the same data as used in this paper. Compared to studies from the United States, studies of European countries have generally reported weaker associations between political orientation and climate change views (McCright and Dunlap, 2011; McCright et al., 2015; Lewis et al., 2019). For example, Smith and Mayer (2018) report that the association between party affiliation, mapped on a left-right continuum to allow for comparisons across countries, and perceived danger from climate change is strongest in English-speaking countries, moderate in non-English-speaking Western European countries, and minimal in post-communist states. McCright et al. (2015) found a similar gap between Western European and former communist countries with regard to the association between political orientation and acceptance of anthropogenic climate change, perceived seriousness, and support for mitigation action.

The ideological differences in climate change concerns that have been identified in previous research may reflect motivated reasoning; a process where existing worldviews and desires influence how individuals interpret available information (Kunda, 1990; Campbell and Kay, 2014; Lewandowsky and Oberauer, 2016). In line with this, the theory of cultural cognition argues that worldviews can make individuals downplay or highlight risks, and generally perceive them differently (Kahan, 2012). These theories have often been used to explain a direct link between political orientation and climate change views, but they could also explain how political orientation may interact with climate change beliefs in shaping perceived risk. While climate skepticism has been found to be higher among right-leaning individuals (McCright et al., 2016) most people in Europe—whether left-leaning or right-leaning—report being at least partly aware of the anthropogenic causes and possible negative consequences of climate change (Steentjes et al., 2017; Pohjolainen et al., 2018). However, political orientation is associated with different values and goals (for a review, see Jost et al., 2009) and thus may direct how information about (the causes and consequences of) climate change are interpreted. While most people seem to acknowledge that climate change will have negative consequences across the world, this could be a more substantial source of concern for left-leaning individuals (usually connected to egalitarian values) as compared to right-leaning individuals (usually connected to individualistic values), as climate change poses a greater threat to the things they value (Steg and Sievers, 2000).

Previous research has shown that political orientation can moderate the relationship between education or self-reported understanding and climate change concern (for reviews, see Hamilton, 2011; McCright, 2011). For example, Hamilton (2008) found that concern about the impacts of climate change on the polar regions increased with higher levels of education for self-reported liberals, while it decreased for those who identified as conservative. Malka et al. (2009) found that higher levels of self-reported knowledge about climate change were related to increased concern among self-identified Democrats, while this was not the case for self-identified Republicans. Similarly, Guber (2013) found that party polarization regarding worry about climate change increased with a higher self-reported understanding of climate change. These studies indicate that individuals might filter information in a way that aligns with their ideology (McCright, 2011). However, this line of research has measured knowledge by asking respondents to indicate their subjective level of understanding, without tapping into the actual content of the knowledge. Sinatra and Seyranian (2015) argue that one can differentiate between unjustified beliefs and justified true beliefs (supported by scientific evidence and justified as knowledge). Neither self-reported understanding nor education necessarily means that the respondents hold justified true beliefs (knowledge) about climate change. While scientific information and education can shape beliefs, people’s climate change beliefs might still differ from the scientific consensus. In the current paper, we focus on the interaction between political orientation and people’s beliefs about the causes and consequences of climate change. While both left-leaning and right-leaning individuals might hold justified true beliefs about climate change, we argue that, as a result of motivated reasoning, such beliefs can lead to different reactions depending on a person’s political orientation.

The present study adds to the literature addressing public perceptions of climate change in a European context. We expect that increased confidence in the anthropogenic nature of climate change, belief in negative impacts, and a left-leaning political orientation, are associated with higher levels of worry. In addition to this, we seek to investigate whether the associations between beliefs towards and worry about climate change are contingent on a person’s political orientation. It is well established that left-leaning individuals are more likely to endorse responsibility for the environment as a moral value (Feinberg and Willer, 2013) and to have concerns about the consequences environmental problems can have on other human beings and on the natural environment itself (Swami et al., 2010). We assume that such differences in values and worldviews may influence to what extent the anthropogenic causes and global consequences of climate change are deemed important for people’s risk perception. Consequently, we expect a stronger relationship between climate change beliefs and worry for left-leaning than for right-leaning individuals. Accounting for possible cross-national differences, we expect the association between political orientation and worry about climate change to be stronger in Western Europe than in post-communist countries (Poortinga et al., 2019).

## Materials and Methods

### Data Collection

This study utilizes data from Round 8 of the European Social Survey (2016). The data were collected in 2016–2017 through face-to-face interviews with *N* = 44,387 respondents from Israel and 22 European countries. Representative samples of the population aged 15+ years were drawn from each country, using strict random probability sampling. The mean age of the overall sample was 46.97 (SD = 18.85), with 48% males (*n* = 24,916) and 52% females (*n* = 27,226) when adjusted for post-stratification and population size weights. The items used in the analysis were taken from the core “Politics” module, as well as the rotating module on “Climate Change and Energy” that was included for the first time in Round 8 of the ESS. For more information on the data, see the documentation report (European Social Survey, 2018).

### Measurements

The dependent variable of the analyses was self-reported worry about climate change, measured with one item. The respondents were asked to answer the question “How worried are you about climate change?” with response categories 1 (*Not at all worried*), 2 (*Not very worried*), 3 (*Somewhat worried*), 4 (*Very worried*), 5 (*Extremely worried*). No answer to the question and the category “Don’t know” were set to missing (*n* = 1733).

Two questions were asked to assess people’s climate change beliefs. Beliefs about the causes of climate change were measured by asking “Do you think that climate change is caused by natural processes, human activity, or both?,” with answer categories 1 (*Entirely by natural processes*), 2 (*Mainly by natural processes*), 3 (*About equally by natural processes and human activity*), 4 (*Mainly by human activity*), or 5 (*Entirely by human activity*). No answer and the options “I don’t think the climate is changing” (*n* = 349) and “Don’t know” (*n* = 2153), were set to missing. The variable was treated as continuous and centered around the grand-mean of *M* = 3.42. Expectations about the severity of climate change impacts were assessed by asking “How good or bad do you think the impact of climate change will be on people across the world?,” with an 11-point response scale ranging from 0 (*Extremely bad*) to 10 (*Extremely good*). The response scale was transformed into a dichotomous variable, coded as 0 (*Belief that the impacts will be good or neutral*), including answers from 0 to 5 on the reversed scale, and 1 (*Belief in mostly bad impacts*), including answers from 6 to 10 on the reversed scale^1^. The category “Don’t know” and no answer was set to missing (*n* = 3155). Political orientation was measured by asking respondents: “In politics people sometimes talk of ‘left’ and ‘right.’ Using this card, where would you place yourself on this scale, where 0 means the left and 10 means the right?” The variable was grand-mean centered (*M* = 5.16). A total of 5804 respondents lacked an answer or were in the category “Don’t know,” which were set to missing. The shares of missing observations on the left-right scale variable were considerably higher in post-communist countries in Eastern and Central Europe than in the remainder of the sample. Table 1 shows a correlation matrix for the outcome and the independent variables.

**TABLE 1:** Correlation matrix.

	**Worry about climate change**	**Climate change attribution**	**Climate change impact**	**Political orientation**
Worry about climate change	1.00			
Climate change attribution	0.30**	1.00		
Climate change impact	0.29**	0.24**	1.00	
Political orientation	−0.11**	−0.10**	−0.12**	1.00

**Correlation is statistically significant at the 0.01 level (two-tailed). Weighted with a combination of post-stratification weight and population weight.

Age, education, and gender were included in the model as control variables based on associations found in previous studies (Marquart-Pyatt, 2008; Hornsey et al., 2016; Poortinga et al., 2019). Gender was dummy coded, with 0 referring to male and 1 to female. Age was treated as a categorical variable with 10-year intervals, centered on the grand-mean of *M* = 49.14. Education had seven categories, representing the highest level of completed education in line with the International Standard Classification of Education (ISCED). The categories were 1 (*ES-ISCED I/less than lower secondary*), 2 (*ES-ISCED II/lower secondary*), 3 (*ES-ISCED IIIb/lower tier upper secondary*), 4 (*ES-ISCED IIIa/upper tier upper secondary*), 5 (*ES-ISCED IV/advanced vocational/sub-degree*), 6 (*ES-ISCED V1/lower tertiary education/BA level*), and 7 (*ES-ISCED V2/higher tertiary education/> = MA level*). The variable was grand-mean centered at 4.01. Table 2 shows descriptive statistics for the variables included in the analyses.

**TABLE 2:** Descriptive Statistics for variables in the study.

**Individual-level (*N* = 44 387)**	***M***	***SD***	**Min**	**Max**
Worry about climate change (1 = Not at all worried; 5 = Extremely worried)	3.06	0.94	1	5
Climate change attribution (1 = Entirely by natural processes; 5 = Entirely by human activity)	3.42	0.83	1	5
Climate change impact (0 = Extremely good; 10 = Extremely bad)	6.80	2.19	0	10
Political orientation (0 = Left; 10 = Right)	4.99	2.18	0	10
Age	46.97	18.85	15	100
Gender (Female)	0.52	0.50	0	1
Education	3.78	1.82	1	7
**Country-level** (*N* = 23)				

*All variables are weighted with a combination of post-stratification weight and population weight.*

### Statistical Analysis

Because people within a country tend to share some features, accounting for heterogeneous variance can help to gain a more accurate picture of residuals. Multilevel models (MLM) accomplish this by dividing the residual variance into within and between components (Rabe-Hesketh and Skrondal, 2012; Snijders and Bosker, 2012). We fitted random intercept and random slope models in order to examine the overall association and interactions between climate change beliefs, political orientation, and worry about climate change. The models comprise two levels that represent individuals (Level 1) nested within countries (Level 2) and were fitted by using the mixed command in Stata 15.

Four models were fitted. We started with an unconditional model (Null Model) followed by a random intercept model with individual-level variables (Model 1), a model that included the interactions (Model 2), and a model including a random slope for political orientation (Model 3). In random intercept models the regression coefficients are held constant across all groups (here: countries), while the intercepts are allowed to vary. This is different from random slope models, where the relationship between a predictor and the outcome is also allowed to vary between groups. In the MLM outlined above, it is possible to predict intercepts and slopes for the countries included in the analysis. However, because countries are treated as random variables, the models cannot be used to compare actual results between the countries (Rabe-Hesketh and Skrondal, 2012, pp. 158–160). Instead of drawing inferences for specific countries included in our sample, we seek to generalize the findings to the total population.

Grand-mean centering was preferred to standardization in the main models because it does not affect the regression slopes and residual variances (Hox et al., 2017). We did, however, use standardization in additional models. The effect size measure *R*^2^ cannot be directly applied to MLM. Instead, we calculated the proportional reduction of variance (PRV), which has been recommended to represent the strength of the relationship between variables in MLM (Rabe-Hesketh and Skrondal, 2012; Billett et al., 2014; Lorah, 2018). Interaction effects were plotted and interpreted by using the margins and marginsplot commands in Stata. Survey weights were not used in the MLM in order to keep the models parsimonious and comparable.

## Results

**FIGURE 1:**
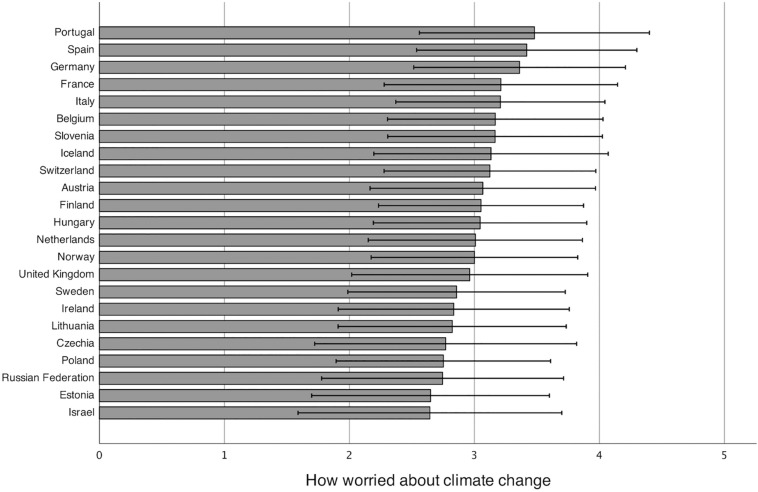
Mean worry by country. Weighted with a combination of post-stratification weight and population weight.

The amount of the variation in worry that is attributable to differences between countries was assessed by fitting a null model without any predictors. The intraclass correlation (ICC) indicated that about 6% of the total variance in individual-level worry about climate change is attributable to variation between countries ICC = 0.06, 95% CI [0.03, 0.10]. The ICC were calculated as the ratio of the country-level variance to the total variance: ICC = σ^2^_country_/(σ^2^_country_ + σ^2^_individual_). Continuing with MLM rather than one-level models is recommended at this ICC level to account for a lack of independence (Bliese, 1998; Hox et al., 2017). The predicted country averages of worry about climate change are shown in Figure 1.

Results from likelihood ratio tests confirmed that the model with individual-level variables (Model 1) has a better fit than the unconditional (‘null’) model χ^2^(6) = 4821.69, *p* < 0.001. Model 1 shows that belief in the anthropogenic nature of climate change and negative impacts on people across the world were associated with more worry, while right-leaning political orientation was associated with less worry (see Table 3).

**TABLE 3:** Model results.

	**Null Model**	**Model 1 (Level 1 variables)**	**Model 2 (interactions)**	**Model 3 (random slope)**
	***B* (SE)**	***B* (SE)**	***B* (SE)**	***B* (SE)**
Fixed coefficients				
Intercept	3.07 (0.05)	2.78 (0.04)	2.77 (0.04)	2.78 (0.04)
Climate change attribution		0.29 (0.01)***	0.29 (0.01)***	0.28 (0.01)***
Climate change impact		0.31 (0.01)***	0.32 (0.01)***	0.32 (0.01)***
Political orientation		−0.03 (0.00)***	−0.01 (0.00)	−0.01 (0.00)
Climate change attribution*political orientation			−0.01 (0.00)**	−0.01 (0.00)**
Climate change impact*political orientation			−0.03 (0.00)***	−0.03 (0.00)***
Age		0.00 (0.00)	0.00 (0.00)	0.00 (0.00)
Gender (Female)		0.12 (0.01)***	0.12 (0.01)***	0.12 (0.01)***
Education		0.02 (0.00)***	0.02 (0.00)***	0.02 (0.00)***
Random parameters (error variance)				
Level 2: Country	0.05 (0.01)	0.03 (0.01)	0.03 (0.01)	0.03 (0.01)
Level 2: Political orientation				0.00 (0.00)
Level 1: Individual	0.77 (0.01)	0.68 (0.01)	0.67 (0.01)	0.67 (0.01)
Log likelihood	−46,111.858	−43,701.011	−43,669.223	−43,658.649
AIC	92,229.72	87,420.03	87,360.45	87,343.3
Variance explained by covariates	ICC = 0.06, 95% CI [0.03,0.10]	Pseudo *R*^2^ = 0.138 *R*_2_^2^ = 0.329 *R*_1_^2^ = 0.126	Pseudo *R*^2^ = 0.140 *R*_2_^2^ = 0.332 *R*_1_^2^ = 0.128	Pseudo *R*^2^ = 0.141 *R*_2_^2^ = 0.334 *R*_1_^2^ = 0.129 ICC = 0.05, 95% CI [0.03,0.08]

*All variables are grand-mean centered, except gender (0 = Male; 1 = Female) and impact (0 = Good; 1 = Bad). ^∗∗^Statistically significant at the *p* = 0.001 level, ^∗∗∗^statistically significant at the *p* < 0.001 level. Total *R*-squared and separate reduction of variance are calculated following the method used in Rabe-Hesketh and Skrondal (2012). *N* = 35,690 individuals, *N* = 23 countries.*

A model including the interaction terms between climate change beliefs and political orientation (Model 2) further improved the fit, χ^2^(2) = 63.58, *p* < 0.001. The interactions between beliefs about climate change causes and political orientation and between expected climate change impacts and political orientation were both statistically significant. As seen in Table 3, the main effect of political orientation was no longer statistically significant after adding the interactions.

The final model (Model 3) included a random slope on political orientation, which again led to an improvement of model fit, χ^2^(2) = 21.15, *p* < 0.001. In this model, increased worry was predicted by belief in anthropogenic climate change and negative impacts. The main effect of political orientation was not statistically significant, just as in Model 2. The interaction between beliefs about climate change causes and political orientation and between beliefs about climate change impacts and political orientation were both still statistically significant. The intercept-slope covariance was not statistically significant, thus including the correlation estimate did not improve the model. The effects of the three control variables age, gender, and education were highly consistent throughout the models. Table 3 shows that age was not statistically significant in any of the models, while higher levels of education were related to increased worry and women were more worried than men across all models.

**FIGURE 2:**
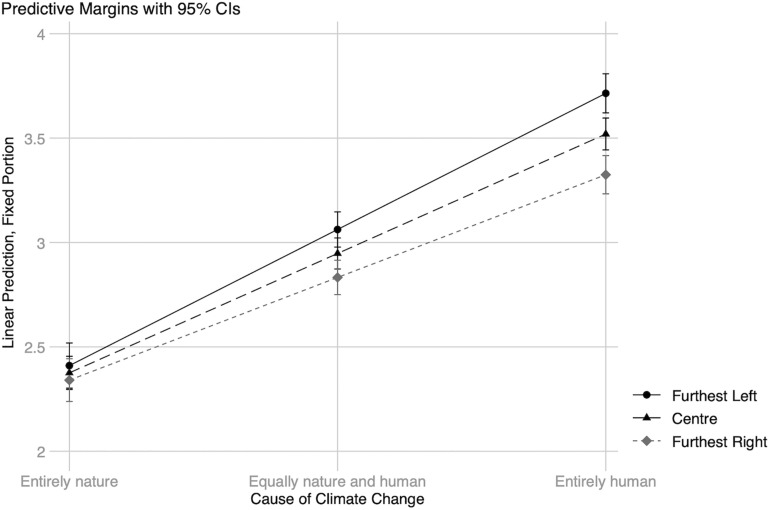
Interaction 1: Climate change attribution*political orientation. Predictive probabilities for increased worry about climate change. The different categories of individual political orientation equal furthest left, center, and furthest right on the 11-point continuum. All other predictors are at their observed values.

The interaction between political orientation and beliefs about climate change causes (see Figure 2) suggests that thinking that climate change is caused by human activity is associated with increased worry, independently of political orientation. The relationship was statistically significant for individuals furthest left (*B* = 0.33 (01), *z* = 22.81, *p* < 0.001 CI [0.30, 0.35]), center (*B* = 0.29 (0.01), *z* = 48.68, *p* < 0.001 CI [0.27, 0.30]), and furthest right (*B* = 0.25 (0.01), *z* = 18.75, *p* < 0.001 CI [0.22, 0.27]) on the spectrum. However, the strongest effect was found for those furthest left, followed by center and furthest right. There were no differences in worry between the three groups for individuals who believe climate change is caused entirely by natural processes.

**FIGURE 3:**
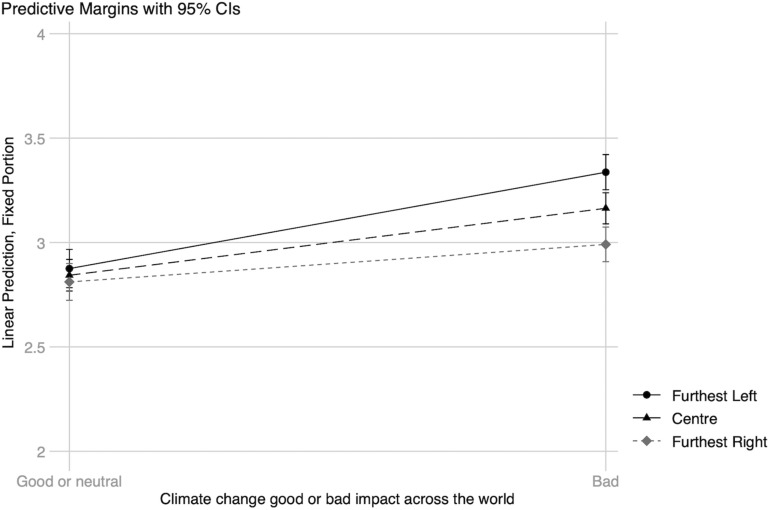
Interaction 2: Climate change impact*political orientation. Predictive probabilities for increased worry about climate change. The different categories of political orientation equal furthest left, center, and furthest right on the 11-point continuum. All other predictors are at their observed values.

The interaction between political orientation and climate change impacts (see Figure 3) indicate that there is no difference in worry between people who place themselves on the political scale furthest to the left, center, or furthest to the right if they believe that the impacts of climate change will be neutral or mostly good. In contrast, worry increased for all three groups when individuals believe that the impacts will be negative across the world, and a gap between the political positions becomes apparent. The positive relationship between belief in negative impacts and worry about climate change was largest for people furthest to the left (*B* = 0.46 (0.03), *z* = 17.58, *p* < 0.001, CI [0.41, 0.51]), followed by people in the center (*B* = 0.32 (0.01), *z* = 31.61, *p* < 0.001, CI [0.30, 0.34]), and smallest for people furthest to the right (*B* = 0.18 (0.02), *z* = 7.65, *p* < 0.001, CI [0.13, 0.23]).

**FIGURE 4:**
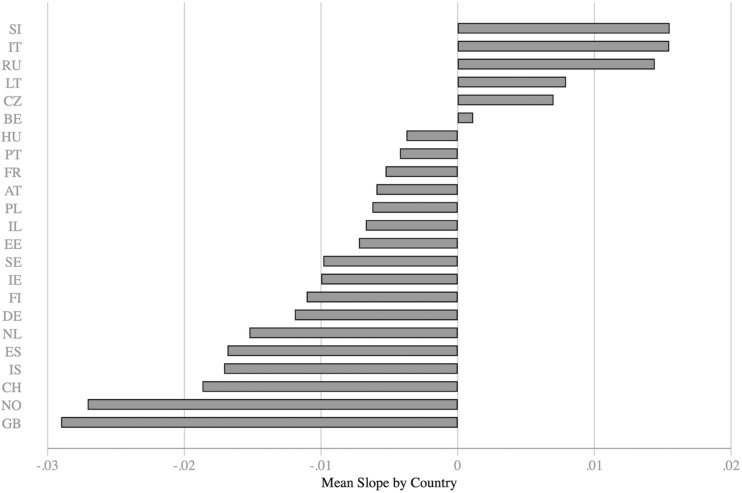
Predicted average slope of political orientation on worry about climate change for each country combined with country-specific intercepts. Austria AT, Belgium BE, Switzerland CH, Czech Republic CZ, Germany DE, Estonia EE, Spain ES, Finland FI, France FR, United Kingdom GB, Hungary HU, Ireland IE, Israel IL, Iceland IS, Italy IT, Lithuania LT, Netherlands NL, Norway NO, Poland PL, Portugal PT, Russian Federation RU, Sweden SE, Slovenia SI.

Figure 4 shows the predicted slopes for political orientation across countries, with all covariates included in the model. The random slopes can be thought of as an interaction between individual-level political orientation and country. The figure shows that the effect of right-leaning political orientation on worry is negative across most countries, but the strength of the effect varies. Consistent with our expectations, the relationship is generally stronger for Western European countries compared to post-communist states. In Italy and some of the post-communist countries, the slopes indicate a positive relationship.

In order to get comparable effect sizes, we refitted Model 1 with standardized versions of the predictors. Standardization was done following recommendations for calculating effect sizes for fixed effects in MLM (Lorah, 2018). The standardized coefficient for climate change causation was β = 0.23 (*p* < 0.001), for belief in negative impacts β = 0.14 (*p* < 0.001), and for political orientation β = −0.06 (*p* < 0.001), education (β = 0.05, *p* < 0.001), and gender (β = 0.06, *p* < 0.001). The same pattern was found in the final model (Model 3), where the standardized coefficient for climate change causes were β = 0.23 (*p* < 0.001), for belief in negative impacts β = 0.14 (*p* < 0.001), and for political orientation β = −0.08 (*p* < 0.001), education (β = 0.04, *p* < 0.001), and gender (β = 0.06, *p* < 0.001). In addition to standardization, effect sizes were indicated by the PRV for each predictor, PRV = (var_model excluding one predictor_ − var_model including all predictors_)/var_model excluding one predictor_ (Billett et al., 2014). The results from refitting Model 1 three times, each time excluding one of the main predictors, indicated that beliefs about climate change causes had the biggest impact out of the covariates (PRV = 0.07), followed by belief in negative impacts (PRV = 0.03). Political orientation did not have a measurable distinct direct contribution. The same results was found for Model 3.

The overall PRV is here referred to as Pseudo *R*^2^ and calculated by following the recommendations from Rabe-Hesketh and Skrondal (2012). By first comparing the unconditional model with Model 1, we see that the covariates in total explained about 14% of the variance in worry about climate change (Total Pseudo *R*^2^ = 0.138). The final model (Model 3), including interactions and a random slope, still explained approximately 14% of the variance (Total Pseudo *R*^2^ = 0.141).

In the final model, about 5% of the variance in worry was attributable to differences between countries ICC = 0.05, 95% CI [0.03, 0.08] compared to 6% in the Null Model ICC = 0.06, 95% CI [0.03, 0.10]. This means that compositional differences in the individual-level variables explained only a small amount of Level 2 variance.

## Discussion

This study examined the overall association between belief in anthropogenic climate change, impact evaluations, political orientation on the one hand and worry about climate change on the other. Because climate change risk perception might be influenced by country-contexts, we further explored possible group effects. Our results indicate that differences between countries explain a relatively small proportion of worry about climate change. One reason might be that there is too much variation within countries to reveal strong contextual effects. For example, prior research has found regional differences in actual and perceived vulnerability to climate change impacts, such as flooding (Brody et al., 2008). This implies that smaller areas, such as municipality or city, might be more suitable to account for possible cluster differences.

In line with prior studies (Lee et al., 2015; Shi et al., 2016), our results indicate that recognizing the human causes of climate change predicts worry. This could be because risks perceived to be human-caused are associated with greater feelings of moral responsibility compared to naturally occurring risks (Böhm and Pfister, 2017). According to the standardized coefficients and the PRV, beliefs about climate change causes had the largest effect out of the predictors. The second largest effect was that of belief in negative impacts. The results showed a positive relationship between belief in negative impacts of climate change and reported levels of worry, which supports prior findings (Böhm and Pfister, 2005; Mayer and Smith, 2019). Research conducted on the concept of psychological distance has indicated that asking about impacts “on people across the world” can have a weaker relation to worry compared to questions about impacts that are geographically and socially close (Spence et al., 2012). It is thus possible that the strength of the relationship between belief in negative impacts and worry about climate change would have been stronger if the question had been framed differently.

Moreover, the predictive power of climate change beliefs on worry was substantially stronger compared to differences in political orientation. The fact that the strength of the association between political orientation and worry differs across countries is likely a partial explanation for the weaker main effect. It should be noted that even though the effect varies, the results indicate that the direction of the relationship is consistent across most countries. Specifically, individuals located further right on the political spectrum generally report being less worried than those further to the left. The reason for the cross-national variation could be explained with the anti-reflexivity thesis, which can also explain why political orientation seems to have more predictive power in the United States (McCright, 2011; McCright et al., 2016) compared to Europe (McCright et al., 2015; Smith and Mayer, 2018; Poortinga et al., 2019).

The anti-reflexivity thesis, often used to explain climate change skepticism, upholds that right-leaning individuals, organizations, and political parties seek to defend the capitalist system, which can be threatened by the need for climate change mitigation (McCright, 2016). Conservative think tanks and anti-reflexivity movements have been especially visible and robust in the United States (McCright et al., 2016). A consequence of these movements could be perceptions of weaker social and scientific consensus concerning the causes and possible consequences of climate change. This is important because prior research indicates that perceived consensus can reduce the gap in reported worry about climate change between the political left and right. For example, Goldberg et al. (2019) found that the relationship between conservative leanings and self-reported worry was, while still existent, substantially less negative for individuals that reported high social consensus among family and friends. Similar results have been found for perceived scientific consensus (Van der Linden et al., 2019).

The fact that the direct association between political orientation and worry is no longer statistically significant when the interactions are present indicates that, rather than it having a direct influence, political orientation alters the relationship between climate change beliefs and worry. Our results show that belief in anthropogenic climate change and its negative impacts on people across the world is more strongly related to worry for left-leaning individuals than for right-leaning individuals. The differences are in line with motivated reasoning, and are plausible when considering typical interests, values, and worldviews within left-leaning versus right-leaning political orientation (Jost et al., 2003; Jost et al., 2009; Balliet et al., 2018). For example, asking specifically for possible *worldwide* consequences of climate change could prime egalitarian values often related to the political left, as opposed to the more individualistic values found on the political right. Consequently, left-leaning individuals might emphasize global risks more than those furthest right on the political spectrum. In line with this, Hart and Nisbet (2012) found that messages that include social distance cues can increase polarization in policy preferences. While positive attitudes toward climate change mitigation policies were independent of whether the potential victims were local or foreign for self-identified Democrats, high social distance reduced policy support among self-identified Republicans. It is important to mention that, though somewhat weaker, the relationship between climate change beliefs and worry are still positive also for right-leaning individuals. Further, there are no differences between the political groups for individuals that believe either that the causation of climate change is entirely natural, or that the impacts will be neutral or good.

Future research including political orientation may need to consider more closely what is meant by “left” and “right” because the effect of political orientation might depend on what these labels represent. The meaning of the labels may vary across countries and even across different groups within countries, and they may signify variation on different dimensions. For example, Caughey et al. (2019) distinguish between economic, social, and immigration-related conservatism and progressivism in Europe, and find that on average, citizens of Northern Europe tend to be more progressive (left-leaning) on immigration and social issues but more conservative (right-leaning) on economic issues than their Southern and Eastern European counterparts. The cross-country difference found in the present study, and previously by Smith and Mayer (2018) and McCright et al. (2015) may thus in part relate to the fact that the left-right scale structures party competition in different countries in different ways. More specifically, the difference between post-communist countries and other democracies may be due to a potentially weaker role of ideological debate along a left-right scale. The data on the share of respondents positioning themselves on a left-right scale suggest a somewhat lower relevance of the left-right scale in Eastern European countries than in Western Europe (Dalton et al., 2011). This is an important limitation because whether respondents think of left and right in economic rather than social terms may matter for the effect of this construct on their perceptions about climate change.

Some other limitations of the current study should be noted, especially in terms of measurement. First, the reported analyses employed single-item measures for the investigated constructs, which can influence their validity. From a theoretical perspective, worry is a personal emotional reaction to a perceived threat and should motivate behavior aimed at reducing the risk (Smith and Leiserowitz, 2014; Van der Linden, 2017). Our findings do not provide any details about what aspects or impacts of climate change people worry about, and whether this differs within or across countries. Previous research has shown that group membership can influence perceptions of environmental issues (Song et al., 2020) and it is likely that left-leaning and right-leaning individuals may worry about different threats. Further, the data provide no insights into how individuals prioritize the issue of climate change compared to other societal issues. Studies using open-ended and unprompted questions to investigate the relative importance of climate change have found climate change to have a relatively low relevance compared to other issues, and that its importance differs across countries (Steentjes et al., 2017). An unspecific understanding of what it means when people say they are “worried” about climate change may limit the practical relevance of the relationship between climate change beliefs and worry. Finally, since the reported analyses were based on cross-sectional data, interpretations about causal directions of the identified relationships have to be made with caution. For example, the relationship between climate change beliefs and worry could be spurious, with a confounding factor explaining their association. Furthermore, while the current paper assumes that climate change beliefs affect worry, it is also possible that worry affects climate change beliefs. Worrying about climate change may stimulate information seeking (Mead et al., 2012) which could increase knowledge about the causes and impacts of climate change. We thus see the investigation of the content of people’s worries and the direction of the relationship between beliefs and worry as fruitful avenues for future research.

## Conclusion

The current study finds that political orientation alters the association between climate change beliefs and worry. Specifically, believing that climate change is caused by humans and will have negative impacts across the world is a more potent source of worry for left-leaning than for right-leaning individuals. The findings might help inform strategies for international climate communication. While focusing on more knowledge and acceptance of anthropogenic climate change remains an important factor across European countries, relying solely on an increase in information is likely not the most effective measure. Instead, communication efforts should take into account that political orientation might influence how beliefs about the causes and consequences of climate change relate to worry. One way to deal with this is to consider relevant worldviews and values within different political orientations and tailor messages accordingly. For example, if the goal is to target individuals with a right-leaning political orientation, focusing on the possible economic or local consequences of climate change might work better than global framings. Further, such climate change information might be more effective if communicated and supported by diverse political elites and advocacy groups (Brulle et al., 2012). Previous research suggests that perceived consensus is highly relevant and that messages about social and scientific consensus can affect worry both directly and indirectly through increased confidence in the anthropogenic character of climate change (Van der Linden et al., 2015, 2019).

## Data Availability Statement

Publicly available datasets were analyzed in this study. The data can be found here: https://www.europeansocialsurvey.org/data/download.html?r=8.

## Ethics Statement

The studies involving human participants were reviewed and approved by ESS ERIC Research Ethics Committee (REC). In accordance with the ESS ERIC Statutes (Article 23.3), the ESS ERIC subscribes to the Declaration on Professional Ethics of the International Statistical Institute. Written informed consent to participate in the study was given by all participants, and was provided by the participants’ legal guardian/next of kin if the respondent was under 16 years of age at the time of the interview.

## Author Contributions

WP and GB were part of the team that designed the climate and energy module of European Social Survey Round 8. TG performed the data analysis and wrote the first draft of the manuscript. All authors contributed to the conception and design of the data analysis and the writing and revisions of the manuscript, and read and approved the final manuscript.

## Conflict of Interest

The authors declare that the research was conducted in the absence of any commercial or financial relationships that could be construed as a potential conflict of interest.

## Footnotes

1 Belief about climate change impacts was dichotomized as this study aimed to distinguish those who think that climate change will have mainly negative impacts from those who believe in either neutral or mainly positive impacts.
